# Notoginsenoside R1 relieves the myocardial infarction via activating the JAK2/STAT3 signaling pathway in vivo and in vitro

**DOI:** 10.1080/21655979.2022.2037366

**Published:** 2022-03-09

**Authors:** Hai Xu, Xiwen Zhang, Yafei Shi, Kun Yu, Yicheng Jiang

**Affiliations:** Department of Cardiology, The First Afliated Hospital of Nanjing Medical University, Huaian City, Jiangsu Province, China

**Keywords:** Myocardial infarction, notoginsenoside R1, JAK2/STAT3, hypoxia/reoxygenation

## Abstract

Myocardial infarction (MI), caused by continuous ischemia and hypoxia of the coronary artery, is one of the major causes of human mortality. This study aimed to investigate the role of notoginsenoside R1 (NGR1) in MI therapy. *In vitro* and *in vivo* models of MI were established by hypoxia/reoxygenation (H/R)-treatment of H9C2 cells and through the ligation of the left anterior descending coronary artery of rats, respectively. CCK-8 and EdU assays were performed to measure cell viability and proliferation, respectively. Flow cytometry and terminal deoxynucleotidyl transferase dUTP nick end labeling (TUNEL) staining were performed to determine the apoptotic rate of cells. Western blot was used to determine protein expression. The MI area was analyzed by 2,3,5-triphenyltetrazolium chloride (TTC) staining. NGR1 promoted viability and proliferation, and inhibited the apoptotic rate of H/R-treated H9C2 cells. In addition, NGR1 downregulated the protein expression of caspase-3 and Bax, and upregulated Bcl-2 expression in H/R-treated H9C2 cells. The JAK2/STAT3 signaling pathway was activated following NGR1 treatment *in vivo* and *in vitro*, and inhibition of the JAK2/STAT3 signaling pathway reversed the effects of NGR1 on H/R-treated H9C2 cells. Finally, NGR1 reduced the area of MI. NGR1 relieved MI *in vivo* and *in vitro* by activating the JAK2/STAT3 signaling pathway.

## Graphical abstract

**Figure:**
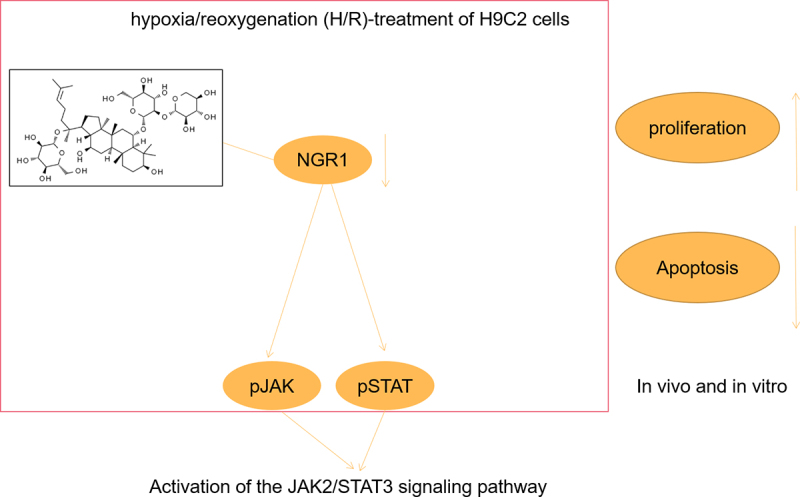

## Introduction

Myocardial infarction (MI), caused by persistent ischemia and hypoxia of the coronary artery, is a major cause of human fatality. Typically, MI results in myocardial ischemia and necrosis, frequently complicated by manifestations such as malignant arrhythmia, heart failure, and shock [1]. MI has a high incidence and mortality rate in most developed countries and is considered a significant health challenge [2]. Growing evidence has revealed the relevance of apoptosis in the pathogenesis of MI, which is closely related to the regulation of myocardial function and heart failure [3,4]. A previous report confirmed that suppressing the apoptotic rate of myocardial cells relieves MI. However, the specific mechanism underlying apoptosis in MI remains unclear. Janus kinase 2/signal transducer and activator of transcription 3 (JAK2/STAT3) is a crucial intracellular signal transduction pathway involved in cell proliferation and differentiation, apoptosis, cellular immunity, inflammation, tumors, and other pathological and physiological processes [5]. STAT3 is the target protein of JAK2, which is typically located in the cytoplasm. Following stimulation by an appropriate signal, JAK2 located on the cell membrane surface is activated via phosphorylation, which in turn activates STAT3 to form homo/heterodimers. The dimer then translocates into the nucleus and combines with the target gene promoter, thus exerting various biological effects [6]. Myocardial ischemia, hypoxia, and reperfusion are considered as stressors that stimulate the production of related cytokines, thus activating the JAK2/STAT3 signaling pathway and providing cardioprotection [7]. Therefore, identifying a novel JAK2/STAT3 signaling pathway activator may be a useful strategy for MI therapy.

*Panax notoginseng*, a popular traditional Chinese medicine, is commonly used to prevent and treat cardiovascular diseases in China. Notoginsenoside R1 (NGR1) is a principal active component of *P. notoginseng*, which has anti-inflammatory, antioxidant, and anti-apoptotic properties [8–10]. Recent reports have indicated that NGR1 elicits superior therapeutic effects on the cardiovascular system [11], nervous system [12], organ development [13], and tumor progression [14]. In addition, Sun et al. [15] confirmed that NGR1 improves septic cardiac dysfunction and inflammatory reactions by regulating the PI3K/Akt signaling pathway. However, to the best of our knowledge, the role of NGR1 in MI has not been reported. Thus, the specific mechanisms of NGR1 remain to be elucidated.

Therefore, we established an *in vitro* MI model using hypoxia/reoxygenation (H/R)-treated H9C2 cells in the present study. In addition, we established an *in vivo* model by ligating the left anterior descending coronary artery. We aimed to analyze the *in vivo* and *in vitro* effects of NGR1 on MI using these models. We hypothesized that NGR1 promotes proliferation and inhibits the apoptosis of H/R-treated H9C2 cells, as well as relieves MI *in vivo* by activating the JAK2/STAT3 signaling pathway.

## Material and methods

### Cell culture and treatment

The rat H9C2 cell line was procured from the Cell Bank of the Shanghai Chinese Academy of Sciences (Shanghai, China). H9C2 cells were seeded in Dulbecco’s modified Eagle’s medium (DMEM, Gibco, NY, USA) containing 10% fetal bovine serum (FBS) and cultured in an incubator with 5% CO_2_ at 37°C. Cells were used for further experiments once they reached logarithmic growth phase (growth to approximately 80% fusion state). The cells were then separated into control (CON), H/R, H/R + 20 μM NGR1 (L-NGR1), H/R + 40 μM NGR1 (H-NGR1), and H/R + H-NGR1 + 50 μM JAK2 inhibitor (AG490) (H-NGR1 + AG490) groups. In the CON group, H9C2 cells were cultured in DMEM supplemented with 10% FBS in a 5% CO_2_ incubator at 37°C. In the H/R, L-NGR1, H-NGR1, and H-NGR1 + AG490 groups, H9C2 cells were cultured in DMEM without FBS in a incubator with 37°C, 5% CO_2_, 94% N_2_, and 1% O_2_ for 24 h, and then cultured in DMEM with 10% FBS in a constant-temperature incubator at 37°C with 5% CO_2_ and 95% air for 6 h. The cultured cells were treated with NGR1 (20 μM or 40 μM) and 50 μM AG490 in the same medium for 24 h.

### Cell Counting Kit-8 (CCK-8)

Cell viability was analyzed using the CCK-8 assay. First, the cells in each group were collected and placed in 96-well plates. Next, 10 μL of CCK-8 solution was added, and the cells were cultured for 72 h. A microplate reader was used to read the absorbance (A) at 450 nm. Cell viability was calculated using the following formula: Cell viability = A _treatment group_/A _control group_.

### 5-ethynyl-2-deoxyuridine (EdU) assay

The cells in each group were collected and plated in a 96-well plate (2 × 10^4^ cells/well). The proliferation ability was detected using a Cell-Light™ EdU DNA Cell Proliferation Kit (Ribobio, Guangzhou, China), following the manufacturer’s instructions.

### Flow cytometry

The apoptotic rate of cells was assessed using an annexin V-FITC kit. Briefly, cells (1 × 10^6^ cells/well) were plated in 24-well plates. After washes and resuspension, 5 μL of Annexin V-FITC and 10 μL of PI were added to the cells. Finally, apoptotic cells were measured using a flow cytometer (BD Biosciences, San Jose, CA, USA).

### Animal experiment

The experiment was approved by the ethics committee of the Affiliated Huaian No. 1 People’s Hospital of Nanjing Medical University. Thirty-six male Sprague-Dawley rats (200 g ± 10 g) were obtained from the Animal Center of Nanjing Medical University. The groups were as follows: control group (Sham, n = 6), model group (Model, n = 10), model group + 20 mg/kg NGR1 (L-NGR1, n = 10), model group + 40 mg/kg NGR1 (H-NGR1, n = 10). The *in vivo* MI model was established by ligating the left anterior descending coronary artery. To establish the MI model, anesthesia was induced using isoflurane. Next, the chest of the rat was shaved and cleaned. The thoracic cavity was opened laterally between the 3^rd^ and 4^th^ intercostals of the anterior cardiac region, and the left anterior descending coronary artery was ligated with a 5/0 surgical suture approximately 2 mm below the pulmonary artery cone and the left atrial appendage. In the Sham group, the rats underwent the same procedure but without ligation. The next day, the rats in the L-NGR1 and H-NGR1 groups were administered NGR1 by gavage once daily. The Sham and Model groups were administered the same amount of normal saline by gavage once daily. The rats were sacrificed after four weeks for the in vivo analysis.

### 2,3,5-triphenyltetrazolium chloride (TTC) staining

Following four weeks of test drug/saline administration, the myocardial tissue was dissected and cleaned with precooled 0.9% sodium chloride solution. Then, the myocardial tissue slices were immersed in 1% TTC phosphate buffer solution at 37°C for 30 min and fixed in 4% paraformaldehyde for 24 h. The infarcted tissue appeared white to light in color, whereas the non-infarcted tissue was dark red. The size of the MI area and the proportion of infarcted tissue areas were measured and calculated.

### Terminal deoxynucleotidyl transferase dUTP nick end labeling (TUNEL) staining

Apoptosis in cells and tissues were analyzed using TUNEL staining. The cells in each group were cultured in 6-well plates and then fixed with 4% formaldehyde. After washing twice with phosphate buffer solution, the cells were treated with 0.5% Triton X-100 for 20 min. Finally, the TUNEL working solution was added, followed by incubation at 37°C for 1 h. Apoptotic cells were analyzed using fluorescence microscopy.

### Western blot

Protein was extracted from cells and tissues using radioimmunoprecipitation assay (RIPA) buffer (Beyotime, Shanghai, China), and the concentration was measured using the BCA Protein Assay Kit (Beyotime). The proteins were separated by 10% sodium dodecyl sulfate-polyacrylamide gel electrophoresis (SDS-PAGE), and then transferred to a polyvinylidene fluoride membrane (Millipore). Next, the PVDF membrane was incubated with the primary antibody including Bax (Abcam, ab32503, 1/1000), Cleaved Caspase 3 (Abcam, ab32042, 1/500), Bcl-2 (Abcam, ab182858, 1/1000), GAPDH (Abcam, ab9485,1/2500), pJAK (Abcam, ab138005,1/1000), JAK (Abcam, ab133666, 1/2000), pSTAT3 (Abcam, ab76315, 1/1000) and STAT3 (Abcam, ab68153, 1/1000) at 4°C overnight. The membrane was further incubated with a secondary antibody (Abcam, ab150077, 1:5000) for 1.5 h. Finally, the target proteins on the membrane were visualized with Pierce™ ECL Western blot substrate. Quantitative detection of the Western blots was performed using ImageJ (version 1.48; National Institute of Health, Bethesda, MD, USA).

Enzyme linked immunosorbent assay (ELISA)

The plasma samples of the rats in different groups were collected, and the levels of brain natriuretic peptide (BNP) and N-terminal pro-B type natriuretic peptide (NT-proBNP) were determined by ELISA kits (Shanghai Chuangxiang Biotechnology Co., Ltd, Shanghai, China) based on the information provided by the manufacturer.

### Statistical analysis

Statistical analysis was performed using SPSS (version 20.0; IBM Corp., Armonk, NY, USA). Data are expressed as the mean ± standard deviation. One-way ANOVA was used for comparison between groups. Statistical significance was set at P < 0.05.

## Results

Myocardial infarction (MI), caused by continuous ischemia and hypoxia of the coronary artery, is one of the major causes of human mortality. This study aimed to investigate the role of notoginsenoside R1 (NGR1) in MI therapy. Our study found that NGR1 promoted viability and proliferation, and inhibited the apoptotic rate of H/R-treated H9C2 cells. In addition, NGR1 relieved MI in vivo and in vitro by activating the JAK2/STAT3 signaling pathway.

### NGR1 promotes the proliferative ability of H9C2 cells

**Figure 1.**
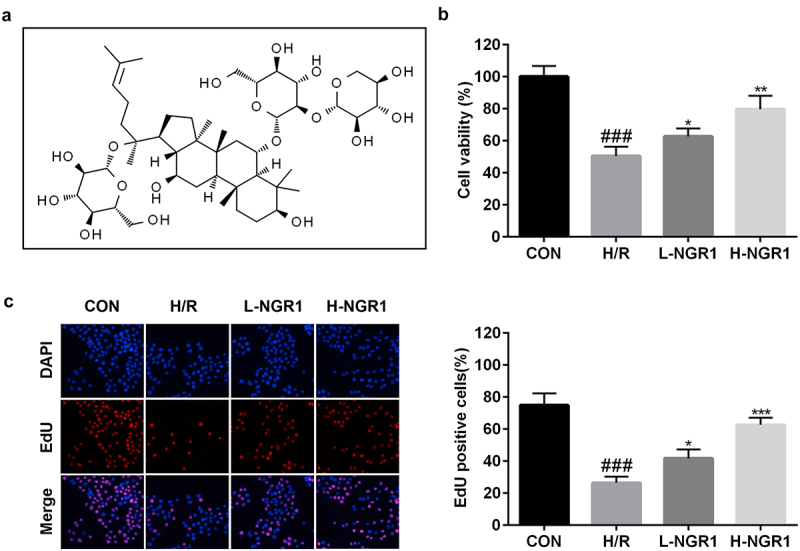
NGR1 promoted the viability and proliferation of the H/R treated H9C2 cells a Molecular structure formula of NGR1. b CCK-8 assay was conducted to detect the cell viability. c EdU assay was performed to measure the proliferation ability of the cells. ^###^P < 0.001, VS CON group. *P < 0.05, **P < 0.01, ***P < 0.001, VS H/R group.

The molecular structure formula of NGR1 was shown in Figure 1(a). The effect of NGR1 on the viability and proliferation of H/R-treated H9C2 cells was measured using CCK-8 and EdU assays, respectively. We observed that NGR1 markedly increased the viability (^###^P < 0.001, *P < 0.05, **P < 0.01) (Figure 1(b)) and proliferation (^###^P < 0.001, *P < 0.05, ***P < 0.001) (Figure 1(c)) of H/R-treated H9C2 cells in a dose-dependent manner.

### NGR1 inhibits apoptosis of H9C2 cells

**Figure 2.**
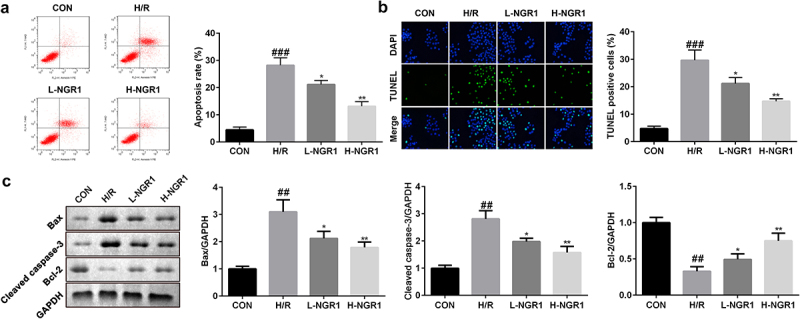
NGR1 inhibited the apoptosis rate of the H/R treated H9C2 cells a-b The apoptosis rate of the cells was measured with Flow cytometry and TUNEL staining. c Western blot was performed to determine the protein expression of Caspase3, Bax and Bcl-2. ^##^P < 0.01, ^###^P < 0.001 VS CON group. *P < 0.05, **P < 0.01, VS H/R group.

Next, we analyzed the effect of NGR1 on H/R-induced apoptosis of H9C2 cells. Flow cytometric analysis revealed that NGR1 markedly decreased the apoptotic rate of H/R-treated H9C2 cells in a dose-dependent manner (^###^P < 0.001, *P < 0.05, **P < 0.01) (Figure 2(a)); this finding was consistent with the results of TUNEL staining (^###^P < 0.001, *P < 0.05, **P < 0.01) (Figure 2(b)). Furthermore, we observed that NGR1 significantly decreased the protein expression of caspase-3 and Bax and increased Bcl-2 expression (^###^P < 0.001, *P < 0.05, **P < 0.01) (Figure 2(c)).

### NGR1 activates the JAK2/STAT3 signaling pathway

**Figure 3.**
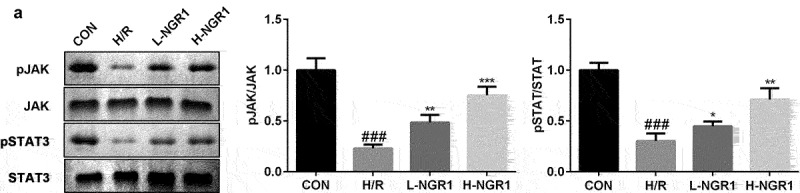
NGR1 activated the JAK2/STAT3 signaling pathway The protein expression of p-JAK2 and p-STAT3 was analyzed by Western blot. ^###^P < 0.001, VS CON group. *P < 0.05, **P < 0.01, ***P < 0.001, VS H/R group.

**Figure 4.**
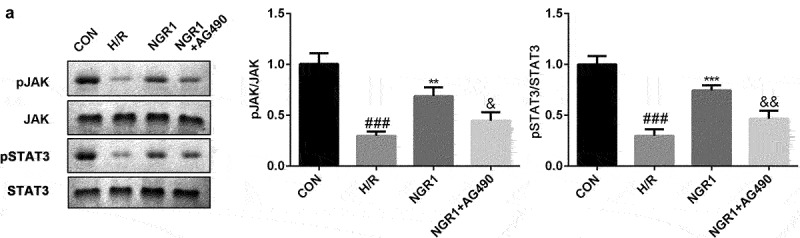
AG490 inhibited the JAK2/STAT3 signaling pathway a The protein expression of p-JAK2 and p-STAT3 was analyzed by Western blot. ^###^P < 0.001, VS CON group. **P < 0.01, ***P < 0.001 VS H/R group. ^&^P < 0.05, ^&&^P < 0.01 VS NGR1 group.

As shown in Figure 3(a), we confirmed that NGR1 promoted the phosphorylation of JAK2 and STAT3 in a dose-dependent manner (^###^P < 0.001, *P < 0.05, **P < 0.01, ***P < 0.001). Additionally, following AG490 treatment, the phosphorylation of JAK2 and STAT3 was decreased compared to that in the NGR1 group (^###^P < 0.001, **P < 0.01, ***P < 0.001, ^&^P < 0.05, ^&&^P < 0.01) (Figure 4(a)).

### Inhibiting the JAK2/STAT3 signaling pathway reverses NGR1 effects

**Figure 5.**
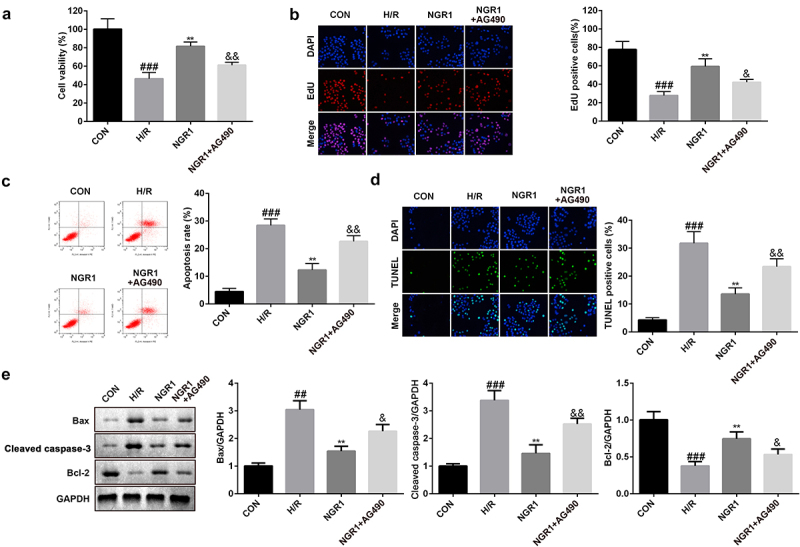
AG490 reversed the effects of NGR1 a CCK-8 assay was conducted to detect the cell viability. b EdU assay was performed to measure the proliferation ability of the cells. c-d The apoptosis rate of the cells was measured with Flow cytometry and TUNEL staining. e Western blot was performed to determine the protein expression of Caspase3, Bax and Bcl-2. ^##^P < 0.01, ^###^P < 0.001, VS CON group. **P < 0.01 VS H/R group. ^&^P < 0.05, ^&&^P < 0.01 VS NGR1 group.

Next, we treated the cells with AG490 to examine the role of the JAK2/STAT3 signaling pathway in MI. The results revealed that following AG490 treatment, cell viability and proliferation were markedly reduced (^###^P < 0.001, **P < 0.01, ^&^P < 0.05, ^&&^P < 0.01) (Figure 5(a,b)), whereas the apoptotic rate was markedly enhanced (^###^P < 0.001, **P < 0.01, ^&&^P < 0.01) (Figure 5(c,d)). Furthermore, AG490 treatment markedly downregulated the protein expression of Bcl-2 and upregulated the expression of caspase-3 and Bax (^###^P < 0.001, **P < 0.01, ^&^P < 0.05, ^&&^P < 0.01) (Figure 5(e)).

### NGR1 relieves MI in vivo

**Figure 6.**
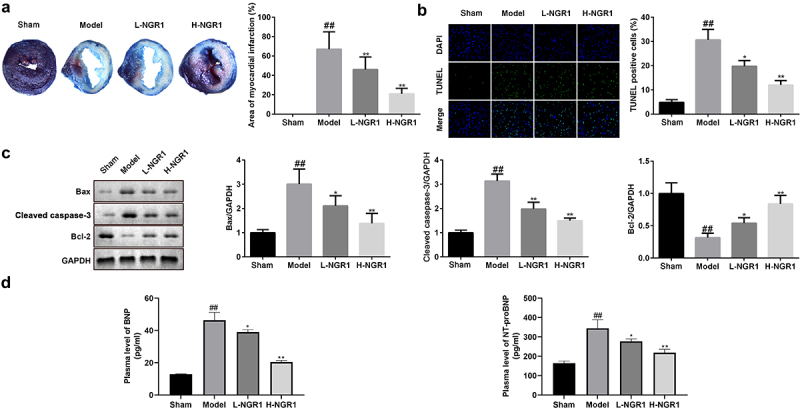
NGR1 relieved the MI in vivo a The myocardial infarction area was tested by TTC staining. b The apoptosis rate of the cells was measured with TUNEL staining. c Western blot was performed to determine the protein expression of Caspase3, Bax and Bcl-2. d ELISA was performed to determine the expressions of BNP and NT-proBNP. ^##^P < 0.01 VS Sham group. *P < 0.05, **P < 0.01, VS Model group.

**Figure 7.**
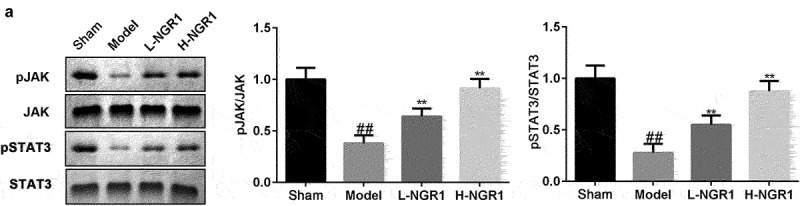
NGR1 activated the JAK2/STAT3 signaling pathway in vivo a The protein expression of p-JAK2 and p-STAT3 was analyzed by Western blot. ^##^P < 0.01 VS Sham group. **P < 0.01 VS Model group.

Finally, we examined the role of NGR1 in the rat MI model. After establishing the MI in vivo model, the 6 rats in the Sham all survived, while 7 of 10 rats in model group, 6 of 10 in model group + 20 mg/kg NGR1 group and 7 of 10 rats in model group + 40 mg/kg NGR1 group survived. TTC staining revealed that NGR1 substantially decreased the MI area in a dose-dependent manner (^##^P < 0.01, **P < 0.01,) (Figure 6(a)). In addition, NGR1 markedly decreased the number of TUNEL-positive cardiomyocytes (^##^P < 0.01, *P < 0.05, **P < 0.01) (Figure 6(b)). Similar to the results in H9C2 cells, the protein expression of caspase-3 and Bax was decreased, whereas that of Bcl-2 was upregulated following NGR1 treatment (^##^P < 0.01, *P < 0.05, **P < 0.01) (Figure 6(c)). Moreover, expression of BNP and NT-proBNP were markedly increased in the model group and significantly decreased by NGR1 treatment in a dose-dependent manner (^##^P < 0.01, *P < 0.05, **P < 0.01) (Figure 6(d)). Furthermore, NGR1 markedly upregulated the phosphorylation of JAK2 and STAT3 in a dose-dependent manner (^##^P < 0.01, **P < 0.01) (Figure 7(a)).

## Discussion

Herein, we confirmed that NGR1 promotes proliferation and suppresses apoptosis in H/R-treated H9C2 cells by regulating the JAK2/STAT3 signaling pathway. In addition, NGR1 relieved the progression of MI *in vivo*. Thus, our findings indicate that NGR1 may be a potential target drug for MI treatment.

MI is a major cardiovascular disease that is closely associated with cardiac insufficiency and sudden cardiac death. Evidence suggests that MI-associated mortality has continued to grow in recent years. MI has been reported to lead to adverse cardiac remodeling and apoptosis in cardiac cells during chronic progression [16]. Since ancient times, Chinese traditional herbs have been widely used to treat cardiovascular diseases. For example, Boarescu et al. [17] found that curcumin has cardioprotective effects and improves cardiac injury after MI. Ling et al. [18] confirmed that resveratrol is involved in cardiac stem cell transplantation via the inhibition of cardiomyocyte apoptosis in an MI rat model. However, studies on NGR1 in MI are limited. Our study confirmed that NGR1 prevents apoptosis *in vivo* and *in vitro* and promotes the growth of H/R-treated H9C2 cells. Likewise, NGR1 was found to inhibit apoptosis in diabetic retinopathy [19], atherosclerosis [10], and burn injury [20]. These findings imply that NGR1 relieves MI progression by suppressing apoptosis. In addition, we analyzed the protein expression of apoptosis-related genes. Caspase-3/Bax/Bcl-2 is an important signaling pathway involved in apoptosis. Caspase-3 is a terminal cleavage enzyme and a marker of apoptosis. Activation of caspase-3 promotes apoptosis. Bax is a pro-apoptotic protein, and Bcl-2 is an anti-apoptotic protein. Under normal physiological conditions, Bax and Bcl-2 exist as Bax/Bcl-2 dimers. Following the upregulation of Bax expression, Bax/Bax homodimerization increases and the caspase family is activated, eventually inducing cell apoptosis. Conversely, overexpression of Bcl-2 promotes the dissociation of Bax/Bax dimer, increases the production of Bax/Bcl-2 dimers, and inhibits cell apoptosis. In the present study, we found that NGR1 downregulated the expression of caspase-3 and Bax, while upregulating Bcl-2 expression *in vivo* and *in vitro*. Zhang et al. [20] demonstrated that 60 μM NGR1 inhibits caspase-3 activation in lipopolysaccharide (LPS)-induced human keratinocyte HaCaT cells. In addition, Luo et al. [21] confirmed that NGR1 upregulates Bcl-2 expression and downregulates Bax expression in the stomach tissues of rats with chronic atrophic gastritis. Our results are consistent with these previous reports. Therefore, our results highlight that NGR1 may prevent cell apoptosis by regulating the expression of apoptosis-related proteins.

The JAK/STAT pathway is mainly composed of the JAK as well as the STAT protein familes. JAK is a common upstream kinase of STAT, and JAK/STAT is widely associated with diverse biological effects such as cell stress, proliferation, differentiation, and apoptosis [6,22,23]. The JAK2/STAT3 pathway is a vital JAK/STAT pathway. Several studies have reported that the JAK2/STAT3 signaling pathway plays a crucial role in cardiomyocyte apoptosis. Hattori et al. [24] demonstrated that activation of the JAK2/STAT3 signaling pathway increases the phosphorylation levels of JAK2 and STAT3, thereby reducing the expression of Bax and caspase-3, and the area of MI, while improving post-ischemic cardiac systolic function. Guo et al. confirmed that matrine relieves myocardial ischemia/reperfusion injury by activating the JAK2/STAT3 signaling pathway. These findings suggest that activation of the JAK2/STAT3 signaling pathway has significant cardioprotective effects. However, whether NGR1 regulates the JAK2/STAT3 signaling pathway has not yet been investigated. In this study, we confirmed that NGR1 promotes the phosphorylation of JAK2 and STAT3 *in vivo* and *in vitro*. We hypothesized that NGR1 may regulate the proliferation and apoptosis of H/R-treated H9C2 cells by activating the JAK2/STAT3 signaling pathway. Accordingly, we inhibited the JAK2/STAT3 signaling pathway using the JAK inhibitor, AG490. Interestingly, AG490 treatment reversed the effects of NGRI on cell proliferation and apoptosis, as well as the protein expression of caspase-3, Bax, and Bcl-2, further indicating that NGR1 attenuates MI by activating the JAK/STAT3 signaling pathway.

## Conclusions

In conclusion, this study is the first to examine the role of NGR1 in the treatment of MI. We demonstrated that NGR1 relieves MI by activating the JAK/STAT3 signaling pathway *in vivo* and *in vitro*. Accordingly, the present study provides a theoretical basis for the application of NGR1 and confirms that NGR1 represents a novel therapeutic drug for MI treatment.

## Research highlights

1. NGR1 promotes the proliferative ability of H9C2 cells
2. NGR1 inhibits apoptosis of H9C2 cells
3. NGR1 activates the JAK2/STAT3 signaling pathway
4. Inhibiting the JAK2/STAT3 signaling pathway reverses NGR1 effects
5. NGR1 relieves MI *in vivo*
